# The Clinical Importance of Cystatin C and Hepatic Artery Resistive Index in Liver Cirrhosis

**DOI:** 10.3390/medicina54030037

**Published:** 2018-05-28

**Authors:** Milos Stulic, Djordje Culafic, Radmila Obrenovic, Goran Jankovic, Tamara Alempijevic, Milica Stojkovic Lalosevic, Natasa Dostanic, Sandra Vezmar Kovacevic, Milica Culafic

**Affiliations:** 1 Clinic for Gastroenterology and Hepatology, Clinical Center of Serbia, School of Medicine-University of Belgrade, 11000 Belgrade, Serbia; djordjeculafic@sbb.rs. (D.C.); goran.jankovic@kcs.ac.rs (G.J.); tamara.alempijevic@med.bg.ac.rs (T.A.); jagodica85@gmail.com (M.S.L.); 2 Institute of Medical Biochemistry, Clinical Center of Serbia, 11000 Belgrade, Serbia; radmilaobrenovic7@gmail.com; 3 Special Hospital for Addiction Diseases “Drajzerova”, 11000 Belgrade, Serbia; nakidostanic@gmail.com; 4 Department of Pharmacokinetics and Clinical Pharmacy, Faculty of Pharmacy-University of Belgrade, 11000 Belgrade, Serbia; svezmar@pharmacy.bg.ac.rs (S.V.K.); milica.culafic@gmail.com (M.C.)

**Keywords:** Cystatin C, hepatic artery resistive index, liver cirrhosis

## Abstract

*Background*: Data suggest cystatin C (CysC) levels and hepatic artery resistive index (HARI) correspond to the progression of chronic liver disease. We aimed to evaluate the clinical significance of these parameters in assessment of fibrosis in patients with liver cirrhosis. *Methods*: The cross-sectional study included 63 patients with liver cirrhosis. A control group consisted of 30 age- and gender-matched healthy persons. *Results*: We confirmed significantly higher values of CysC in patients with cirrhosis compared to control group (*p* = 0.036). Average value of HARI in the examined group was increased (0.72 ± 0.06) and there was the statistically significant difference compared to controls (0.66 ± 0.03) (*p* < 0.001). We found statistically significant correlation between HARI and CysC in the study group. Analyzing the possibility of distinguishing healthy subjects from patients with fibrosis, we have found that the area under the curve is far greater in the HARI index than CysC. Comparison of CysC among Child–Pugh stages and correlation with a model for end-stage liver disease (MELD) score showed statistically significant results. *Conclusion*: We confirmed HARI is a more accurate parameter than CysC in discriminating healthy subjects from patients with fibrosis, while CysC could be a better indicator of the stage of liver cirrhosis.

## 1. Introduction

Liver cirrhosis is the common pathological state of liver damage arising from a wide variety of chronic liver diseases. Even though the causes of liver cirrhosis are known to be multifactorial, degeneration and necrosis of hepatocytes, liver parenchyma replaced by fibrotic tissue and regenerative nodules are pathological characteristics common to all cases of liver cirrhosis [1,2].

Liver cirrhosis is recognized as a leading cause of death with portal hypertension (PHT) being the most common complication of the disease. The most relevant determinant of PHT in cirrhosis is the increased intrahepatic vascular resistance as a consequence of liver vascular architecture distortion and hepatic sinusoidal cellular alterations that promote constriction of the hepatic sinusoids and fibrosis. Furthermore, mechanism contributing to PHT is an increased splanchnic blood flow, in part explained with mesenteric arteriolar vasodilatation and decreased vascular responsiveness to endogenous vasoconstrictors [3,4].

A few studies showed that serum cystatin C (CysC) is increased with the progression of the chronic liver disease and that it might be involved in the pathogenesis of liver fibrosis [5,6]. CysC is a low-molecular weight non-alkaline glycosylated protein, containing 122 amino acids. Nucleated cells can produce sustainable CysC that is secreted into the extracellular fluid, including blood, cerebrospinal fluid and semen. Previous studies reported that CysC is free from a conventional storage environment and not influenced by factors such as gender, age, diet and inflammation [5,7,8].

Doppler ultrasonography is commonly utilized as a first noninvasive diagnostic procedure in patients with liver cirrhosis and portal hypertension. Hepatic artery resistive index (HARI) is a Doppler ultrasonography parameter that is used to follow up microcirculatory resistance in liver damage [9].

Our research aimed to evaluate the clinical significance of CysC and HARI in the assessment of hepatic fibrosis in patients with liver cirrhosis.

## 2. Materials and Methods

We conducted a cross-sectional study of 63 patients, age ≥18 years, with alcoholic (65.1%) or viral liver cirrhosis (hepatitis C viral cirrhosis, 20.6%; and hepatitis B viral cirrhosis, 14.3%) examined and treated between December 2011 and September 2013. The healthy control group comprised of 30 age- and gender-matched subjects. The diagnostic approach for both groups was based on anamnestic data (consumption of alcohol more than 50 g/day over a five-year period except last 6 months), physical examination (presence of clinical stigmata of chronic alcoholism), laboratory tests (hepatocyte integrity, parameters of cholestasis, and synthetic liver function) and abdominal Doppler ultrasonography findings. Diagnosis of B viral cirrhosis was based on serology (HBsAg, HBeAg, anti-HBe and anti-HBc IgG) and determination of polymerase chain reaction hepatitis B virus deoxyribonucleic acid (PCR HBV DNA). Hepatitis C viral cirrhosis was verified by the presence of anti-HCV antibodies and quantification of viral load per milliliter of blood by polymerase chain reaction hepatitis C virus ribonucleic acid (PCR HCV RNA). All patients had abdominal Doppler ultrasonography and upper endoscopy performed during routine work up.

The degree of liver insufficiency was assessed according to the Child–Pugh classification into three stages: A (36.5%), B (33.3%) and C (30.2%) (score A ≤ 6, B 7–9, C ≥ 10) [10]. The diagnosis of hepatic encephalopathy was based on clinical criteria. The West Haven Criteria for mental status grading is applied to assess the severity of hepatic encephalopathy [11]. Model for end-stage liver disease (MELD) score was also used to evaluate patients with liver cirrhosis [12].

Exclusion criteria we applied were as follows: presence of hepatocellular carcinoma, gastrointestinal bleeding, hepatorenal syndrome, and any superimposed conditions such as infection, intrinsic renal disease, chronic obstructive pulmonary disease, congestive heart failure, thyroid dysfunction, and diabetes mellitus. Patients receiving corticosteroids, antiviral agents, angiotensin II receptor blockers, angiotensin**-**converting enzyme inhibitors, nonsteroidal anti-inflammatory drugs, beta blockers, nitrates and amino acids L-arginine and L-ornithine were also excluded from the study.

### 2.1. Biochemistry

Venous blood samples were collected in vacutainers without additives, centrifuged at 3500 rpm (≈2000 g) and preserved at minus 80 °C after separation. The PENIA method (Particle-Enhanced Nephelometric Immuno-Assay) was used to determine CysC serum concentration, measured with the SIEMENS (Marburg, Germany) tests, on a laser nephelometer (BN IIDadeBehring). CysC referent value was 0.59–1.04 mg/L. 

### 2.2. Abdominal Doppler Ultrasonography 

The liver size, echo structure of the hepatic parenchyma and possible focal changes, spleen diameter, and presence of ascites were examined with ultrasonography (Toshiba Core Vision, with Doppler duplex convex probe, 3.5 MHz). Color Doppler duplex ultrasonography was used to determine HARI, portal vein diameter and blood flow velocity. All ultrasonographic findings were made by the same person. Portal vein diameter less than 13 mm and blood flow velocity ranging from 16–31 cm/s in fasting adults were considered as normal. Resistive index (RI) equals peak systolic velocity minus the final diastolic velocity divided by the peak systolic velocity. RI below 0.7 is considered normal [13,14].

### 2.3. Upper Endoscopy

Panendoscopy (Olympus exera II CV-165, type Q165) was performed to examine signs and consequences of portal hypertension: the existence of esophageal varices, gastric varices and portal hypertensive gastropathy (PHG). When esophageal varices were present, their size was graded as I–IV using Paquet grading system [15].

### 2.4. Statistical Analysis

Categorical data are presented as number (percentage), and continuous data as mean ± standard deviations or median (25th–75th percentile). Group comparisons were performed using t test or Mann–Whitney U test for continuous data (depending on data distribution) and chi-square test for nominal data. Continuous data distribution was examined using the Shapiro–Wilks test. Spearman’s correlation analysis was performed to evaluate the relationship between continuous variables with non-normal distribution. Receiver operating characteristics (ROC) and area under the curve (AUC) were used to assess diagnostic ability of continuous variables in discriminating healthy patients from patients with cirrhosis. All data were analyzed using SPSS 20.0 (IBM Corporation) statistical software. All *p* values less than 0.05 were considered significant.

### 2.5. Ethical Considerations

The study was conducted in accordance with Guidelines for Good Clinical Practice, the Declaration of Helsinki, and local laws and regulations. The protocol was approved by joint Research and Ethics Committee of the Clinical Center of Serbia, Belgrade, filed under number 2385/5. Written informed consent was obtained from all the participants in the study.

## 3. Results

Basic demographic data of our patients were presented earlier [16] (Table 1). The average diameter of the portal vein, HARI and CysC were significantly larger in the study group, while blood flow velocity was significantly slower (Table 1).

**Figure 1:**
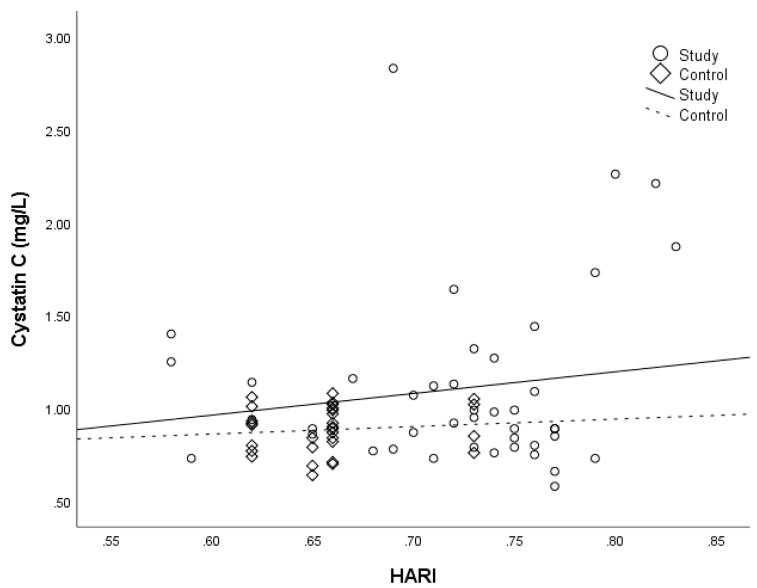
HARI and cystatin C: study vs. control. HARI, hepatic artery resistive index.

We did not identify any correlation between diameter and blood flow velocity of the portal vein with levels of CysC in the study group (Table 2). However, we found a statistically significant correlation between HARI and CysC (Table 2 and Figure 1).

Moreover, increased values of HARI were detected in 46 (73%) patients with an average value of 0.75 ± 0.03, and in this patient group, we also noted a strong positive correlation with levels of CysC (r_s_ = 0.530, *p* < 0.001). In this group, there were 31 (67.4%) patients with alcoholic liver cirrhosis and 15 patients (32.6%) with viral cirrhosis. The analysis of these two subgroups of patients did not determine a statistically significant difference in HARI values.

However, there were no statistically significant differences between values of diameter and blood flow velocity of the portal vein and HARI among Child–Pugh stages and no correlation with MELD score. Comparison of CysC among Child–Pugh stages and correlation with a MELD score showed statistically significant results, which were presented earlier in our article [16].

**Figure 2:**
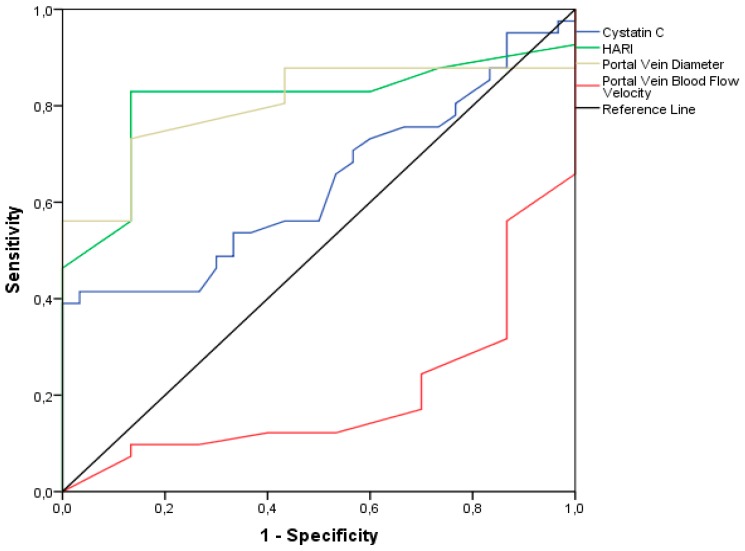
Receiver operating characteristics (ROC) and area under the curve (AUC) of CysC and Doppler ultrasonography findings. HARI, hepatic artery resistive index.

Analyzing the possibility of distinguishing healthy subjects from patients with fibrosis, we have found that the area under the curve is far greater in the HARI index than CysC (Table 3 and Figure 2). 

Esophageal varices were detected in 51 (80.9%) patients. There were 13 patients with grade I esophageal varices, 23 had grade II esophageal varices, and 15 patients had grade III varices. Nine (14.3%) patients also had gastric varices, and 47 (74.6%) had concomitant PHG. Patients with esophageal varices had statistically higher values of CysC when compared to patients without varices (*p* = 0.048). Values of CysC did not differ significantly among patients with different grades of esophageal varices (*p* = 0.117).

When the presence of esophageal varices was compared to Doppler ultrasonography findings, our results showed no statistically significant correlation with portal vein diameter (*p* = 0.756), blood flow velocity (*p* = 0.125) or HARI (*p* = 0.944). In addition, there was no statistically significant difference between HARI values in patients with and without PHG (*p* = 0.153).

## 4. Discussion

Our study supports the evidence of considering Doppler ultrasonography findings with CysC levels when staging liver damage. As the results suggest, HARI should be monitored closely when fibrosis is suspected while CysC could help to identify cirrhosis stage, before considering liver biopsy as an invasive approach to confirm the diagnosis. 

Portal hypertension promotes hyperdynamic circulation and portosystemic collateral vessels, which can lead to the development of esophageal and gastric varices that are responsible for life-threatening consequences such as gastro-esophageal bleeding [17].

Duplex ultrasonography represents the best noninvasive technique for assessing a splanchnic circulation of patients with liver cirrhosis [18]. If correctly performed, duplex Doppler ultrasonography is at least as accurate as angiography. Because of the wider diameter of the portal vein as well as the lesser dependence on respiratory movements, errors in measurement of portal vein diameter in patients with portal hypertension are minor in comparison with errors in healthy persons. In general, duplex Doppler ultrasonography is an acceptable procedure for clinical monitoring of patients with portal hypertension [19]. It is readily feasible, mainly available in all hepatologic centers and significantly cheaper compared to angiography. The association of Doppler ultrasonography and biochemistry analyses challenges further researcher to identify parameters that correlate the most with non-invasive techniques.

We decided to compare HARI, VPD and VP BFV, as easily available Doppler ultrasonography findings with CysC, a biochemical parameter that is increasingly used in clinical practice and which is topic of numerous studies. We placed the emphasis on HARI and CysC, as relevant parameters for assessing the stage of liver disease, which we have confirmed with the literature data.

In accordance with our results, Shateri et al. showed that the rate of pathological changes in the portal hemodynamic and sonographic verified portal vein diameter and blood flow velocity does not accurately correlate with the deterioration degree of the liver function in patients with liver cirrhosis [20].

There is no consensus on the role of classic Doppler ultrasonography in the evaluation of esophageal varices. A study showed that duplex Doppler ultrasonography is a quantitative technique for the assessment of hemodynamic changes in patients with portal hypertension and appears to be useful in the identification of patients with liver cirrhosis at risk of upper gastrointestinal bleeding [21]. 

Conversely, Zardi et al. reported that the mean portal vein diameter slightly but not significantly increased in patients with PHG. The oscillatory trend of portal vein diameter from control to large size esophageal varices might indicate that they may unload portal pressure in the initial phases of portal hypertension. Moreover, these authors concluded that portal vein diameter was not able to predict presence or size of esophageal varices in a large series of patients with cirrhosis [22]. Shabestari et al. also did not find a statistically significant difference between portal vein diameter and blood flow velocity with the presence or size of esophageal varices [23]. Our results are in accordance with these studies.

In patients with chronic viral hepatitis, HARI was significantly greater in those with moderate or severe inflammation compared to those with mild inflammation or to control subjects, as reported by Haktanir et al. [24]. Furthermore, Piscaglia et al. concluded that HARI of patients with chronic viral hepatitis seems to be significantly influenced by the degree of fibrous tissue deposits. It is essential to disclose the increase of the HARI in mildly inflamed cases in chronic hepatitis B and C virus carriers because this increase can be used as an indicator of disease progression and also for biopsy decision-making [9].

Additionally, a few studies confirmed that HARI is increased in liver cirrhosis, but no significant correlation between the degree of cirrhosis and/or inflammation was noted [25,26].

These studies have shown that HARI is a superior parameter for isolating patients with fibrosis compared to healthy controls. However, by the development of liver cirrhosis, the occurrence of collateral circulation and esophageal varices, which decompresses pressure in the splanchnic blood vessels, all Doppler ultrasonographic findings including HARI lose the ability to assess the stage of the disease. Our results are consistent with those findings. 

Values of CysC are significantly higher in patients with cirrhosis, as we previously reported [16]. Progression of fibrosis in chronic liver diseases results from an imbalance between synthesis and degradation of extracellular matrix that leads to the first increase in serum CysC levels. The fibrogenesis depends on the activity of stimulated hepatic stellate cells, and the degradation is carried out by a series of serine proteases, cysteine proteases and metalloproteinases. CysC is a potent inhibitor of lysosomal cysteine proteases and may act as a potent profibrogenic agent [27].

Transforming growth factor β (TGF β) is the essential cytokine inducing liver fibrogenesis, as it promotes the differentiation of hepatic stellate cells (HSC) in hepatic myofibroblasts. In vitro, TGF β is a potent inducer of CysC secretion in vascular smooth muscle cells [28]. The CysC expression is upregulated in the course of HSC transdifferentiation in myofibroblasts under the stimulus of TGF β [6]. Two studies observed patients with chronic hepatitis C and reported significantly increased plasma TGF β levels parallel with the severity of fibrosis (in line with liver biopsy), but also a decline when reaching the cirrhotic stage [29,30].

A second increase of serum CysC concentration in patients with liver cirrhosis could be interpreted as a decline in glomerular filtration rate [31]. This could be the main reason for the correlation of elevated values of CysC and stage of liver cirrhosis. This study has a certain limitation in regards to sample size. Further research should include more patients, and it would be interesting to analyze these parameters on the same patients over time. 

In conclusion, we confirmed that HARI is a more accurate parameter than CysC in distinguishing patients with fibrosis, while CysC could be a better indicator of the stage of liver cirrhosis. Both parameters could be relevant in every day clinical practice.

## Figures and Tables

**Table 1:** Basic demographic data, Doppler ultrasonography findings and cystatin C in study and control group.

	Study Group	Control Group	*p* Value
Age, mean ± SD, years	50.8 ± 13.5	48.2 ± 17.6	0.434 ^a^
Males, n (%)	47 (74.6)	24 (80)	0.567 ^b^
VPD, mean ± SD, mm	13.51 ± 3.02	10.55 ± 0.96	<0.001 ^a^
VP BFV, mean ± SD, cm/s	26.48 ± 8.38	34.25 ± 5.48	<0.001 ^a^
HARI, mean ± SD	0.72 ± 0.06	0.66 ± 0.03	<0.001 ^a^
CysC, median (25–75th percentile), mg/L	0.97 (0.80–1.20)	0.90 (0.79–1.00)	0.036 ^c^

VPD, portal vein diameter; VP BFV, blood flow velocity of portal vein; HARI, hepatic artery resistive index; CysC, cystatin C; ^a^ T-test, ^b^ Pearson chi-square test, ^c^ Mann–Whitney U test.

**Table 2:** Correlation between Doppler ultrasonography findings and cystatin C.

	CysC			
	Study Group		Control Group	
	r_s_	*p*	r_s_	*p*
VPD	0.052	0.741	0.270	0.149
VP BFV	−0.242	0.123	–0.071	0.710
HARI	0.268	0.038	0.175	0.356

VPD, portal vein diameter; VP BFV, blood flow velocity of the portal vein; HARI, hepatic artery resistive index; CysC, cystatin C; r_s_, Spearman’s rank correlation coefficient.

**Table 3:** The characteristics of CysC and Doppler ultrasonography findings in differentiation healthy controls from patients with fibrosis.

	Area (95% CI)	*p* Value	Cut off Value	Sn	Sp		PPV	NPV	Accuracy
CysC	0.637 (0.509–0.765)	0.050	1.085	38.3	100		100	44.7	58.5
HARI	0.810 (0.703–0.917)	<0.001	0.665	83.7	86.7		90.0	78.8	84.9
VPD	0.803 (0.695–0.910)	<0.001	11.2	73.8	86.6		88.6	70.3	79.2
VP BFV	0.799 (0.693–0.904)	<0.001	29.5	66.6	86.6		87.5	65.0	75.0

CysC, cystatin C; HARI, hepatic artery resistive index; VPD, portal vein diameter; VP BFV, blood flow velocity of portal vein; PPV, positive predictive value; NPV, negative predictive value.
